# Novel blood-based microRNA biomarker panel for early diagnosis of chronic pancreatitis

**DOI:** 10.1038/srep40019

**Published:** 2017-01-11

**Authors:** Lei Xin, Jun Gao, Dan Wang, Jin-Huan Lin, Zhuan Liao, Jun-Tao Ji, Ting-Ting Du, Fei Jiang, Liang-Hao Hu, Zhao-Shen Li

**Affiliations:** 1 Department of Gastroenterology, Changhai Hospital, the Second Military Medical University, Shanghai, China

## Abstract

Chronic pancreatitis (CP) is an inflammatory disease characterized by progressive fibrosis of pancreas. Early diagnosis will improve the prognosis of patients. This study aimed to obtain serum miRNA biomarkers for early diagnosis of CP. In the current study, we analyzed the differentially expressed miRNAs (DEmiRs) of CP patients from Gene Expression Omnibus (GEO), and the DEmiRs in plasma of early CP patients (n = 10) from clinic by miRNA microarrays. Expression levels of DEmiRs were further tested in clinical samples including early CP patients (n = 20), late CP patients (n = 20) and healthy controls (n = 18). The primary endpoints were area under curve (AUC) and expression levels of DEmiRs. Four DEmiRs (hsa-miR-320a-d) were obtained from GEO CP, meanwhile two (hsa-miR-221 and hsa-miR-130a) were identified as distinct biomarkers of early CP by miRNA microarrays. When applied on clinical serum samples, hsa-miR-320a-d were accurate in predicting late CP, while hsa-miR-221 and hsa-miR-130a were accurate in predicting early CP with AUC of 100.0% and 87.5%. Our study indicates that miRNA expression profile is different in early and late CP. Hsa-miR-221 and hsa-miR-130a are biomarkers of early CP, and the panel of the above 6 serum miRNAs has the potential to be applied clinically for early diagnosis of CP.

Chronic pancreatitis (CP) is an inflammatory disease characterized by progressive fibrosis of parenchyma, irreversible impairment of both exocrine and endocrine functions^1^,^2^. The diagnosis of CP is now mainly based on clinical manifestation and image findings. However, CP patients who manifest diabetes, steatorrhea or typical imaging features (atrophic pancreas and pancreatic calculi) have progressed into the late stage of the disease (late CP), and the function of pancreas would not restore significantly despite current medication and endoscopic management^3^,^4^,^5^. Therefore, serum biomarkers or other modalities that can identify CP at early stage (early CP) is essential for diagnosis and management of CP patients.

In recent years, microRNAs (miRNAs) are suggested to be novel potential biomarkers in many diseases and have been recognized as central modulators that regulate inflammation and fibrogenesis^6^,^7^,^8^. Regarding the pancreatic disease, a few studies addressed the miRNA expression profiles of CP. One study enrolled 65 patients with pancreatic cancer and 42 patients with CP, and the miRNA microarray analysis showed that 15 high-expressed miRNAs and 8 low-expressed miRNAs could differentiate CP from pancreatic cancer^9^. Another study reanalyzed the CP-related miRNA expression profile in mice, and found that miR-124a played a significant role in the CP pathogenesis, which may be a target for diagnosis and treatment of CP^10^. However, previous studies did not separate early and late CP and were mainly based on the expression profiles of pancreas tissue, which decreased the application potential in clinical practice.

In this study, we compared the differences of serum miRNA expression in early and late CP patients and healthy controls, aiming to obtain serum miRNA biomarkers for early diagnosis of CP. Moreover, function of these miRNAs and miRNA-miRNA correlation network were analyzed.

## Results

### Analysis of differentially expressed miRNAs (DEmiRs) for Gene Expression Omnibus (GEO) CP

**Figure 1:**
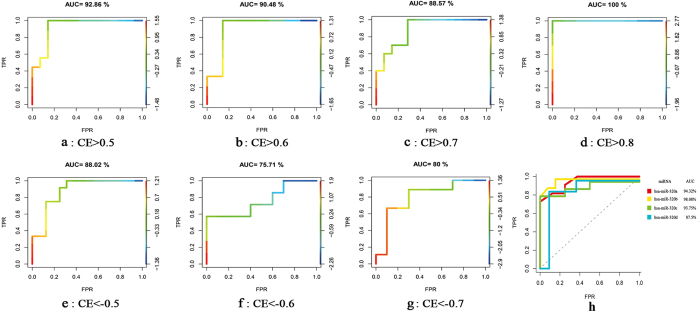
DEmiRs of late CP. A RBF kernel-based SVM was designed and 5-fold cross-validation strategy was utilized to evaluate the performance of SVM classifier. The outcome of prediction was then assessed by ROC curve analysis. (**a**–**g**) The ROC curves of SVM classifier created on miRNA-miRNA pairs with diverse CE values. (**a**–**d**) The positive correlation miRNA-miRNA pairs; (**e**–**g**) The negative correlation miRNA-miRNA pairs; (**h**) The ROC curves of SVM classifier. It was created on 4 features: hsa-miR-320a, hsa-miR-320b, hsa-miR-320c and hsa-miR-320d. The diagonal line was the baseline (GEO CP, n = 38; GEO healthy control, n = 38).

After downloading and analyzing the miRNA expression profile from GEO database, a total of 136 DEmiRs were acquired in the miRNA expression profiles in GEO CP, including 59 up-regulated miRNAs and 77 down-regulated miRNAs. Through the correlation analysis and function enrichment of the DEmiRs, we acquired 45 negative and 115 positive DEmiR-DEmiR pairs. To evaluate the importance in the prediction of GEO CP, the miRNAs in different correlation coefficient (CE) area were selected as the classification features, and the support vector machine (SVM) method was applied to construct the classifier which was trained using 5-fold cross-validation and assessed the effect of classifying (Fig. 1).

The prediction accuracy for miRNA-miRNA pairs in positive correlation (Fig. 1a–d) was higher than in negative correlation (Fig. 1e–g). Moreover, when the CE values of miRNA-miRNA pairs were more than 0.8, area under the curve (AUC) was 100.0% (Fig. 1d). The CE values of hsa-miR-320a/hsa-miR-320b and hsa-miR-320c/hsa-miR-320d were higher than 0.8. To screen the biomarkers of GEO CP, the four miRNAs were further evaluated in predicting GEO CP samples (n = 38) from normal samples (n = 38), and the AUC of hsa-miR-320a and hsa-miR-320b were 94.3% and 98.1% respectively, which were higher than hsa-miR-320c and hsa-miR-320d (Fig. 1h).

According to the performance of SVM classifier for the prediction of GEO CP, which was analyzed by the AUC metric (Fig. 1a–h), hsa-miR-320a-d were closely correlated with each other with high AUC values, indicating that a combination of hsa-miR-320a/b and hsa-miR-320c/d could improve the prediction power for GEO CP.

### MiRNA expression analysis for early CP

**Figure 2:**
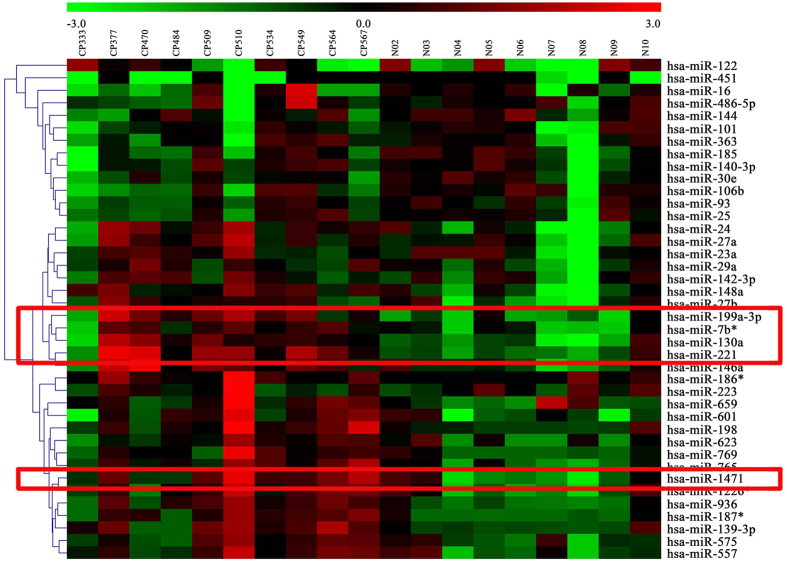
Clustering analysis of miRNA expression values for top 40 miRNAs of early CP. The change of color from green to red represented the change in |logFC| from low to high (early CP, n = 10; healthy control, n = 9). FC was the fold change value. The DEmiRs were framed with red box. The miRNA expression profile was acquired using Agilent miRNA microarrays (G4450AA). The fluorescence intensity signal values were analyzed with software such as Agilent G4450AA Feature Extraction Software 9.5 and Agilent Scan Control Software version A. 7.0 etc.

Agilent miRNA microarrays were utilized to evaluate the expression of miRNAs in early CP. Ten early CP samples and 9 healthy control samples were analyzed. Four DEmiRs (hsa-miR-221, hsa-miR-199a-3p, hsa-miR-130a and hsa-miR-1471) were identified in early CP, all of which were up-regulated (P < 0.05). Hierarchical clustering analysis was performed on the expression values of top 40 miRNAs (Fig. 2).

**Figure 3:**
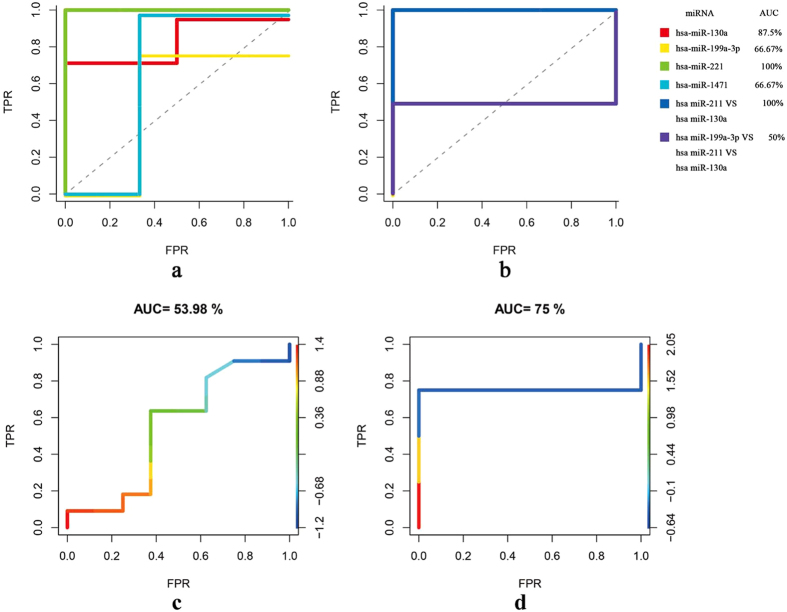
DEmiRs of early CP. A RBF kernel-based SVM was designed and 5-fold cross-validation strategy was utilized to evaluate the performance of SVM classifier. The outcome of prediction was then assessed by ROC curve analysis. (**a**,**b**) The ROC curves of SVM classifier of early CP. (**a**) Every DEmiR as a classification feature; (**b**) The combined DEmiRs were classification features based on the correlation. The diagonal line is the baseline; (**c**,**d**) Comparing the classification effect of biomarkers. c, prediction effect of early CP biomarkers in late CP (GEO CP, n = 38; GEO healthy controls, n = 38); (**d**) prediction effect of late CP biomarkers in early CP (early CP, n = 10; healthy control, n = 9).

The four DEmiRs were further evaluated in the prediction of early CP. The AUC score of hsa-miR-221 was 100.0% and hsa-miR-130a was 87.5% (Fig. 3a). Hsa-miR-221 was highly correlated with hsa-miR-130a with CE 0.9. When acting both of hsa-miR-221 and hsa-miR-130a as classification features simultaneously, the results showed that they could perfectly distinguish the early CP (n = 10) from normal controls (n = 9) with AUC of 100.0% (Fig. 3b).

Subsequently, we applied the two potential biomarkers of early CP to predict GEO CP and four biomarkers of GEO CP to predict early CP. The biomarkers of early CP could not effectively identify GEO CP patients (Fig. 3c), and vice versa (Fig. 3d).

### Expression levels of the DEmiRs

**Figure 4:**
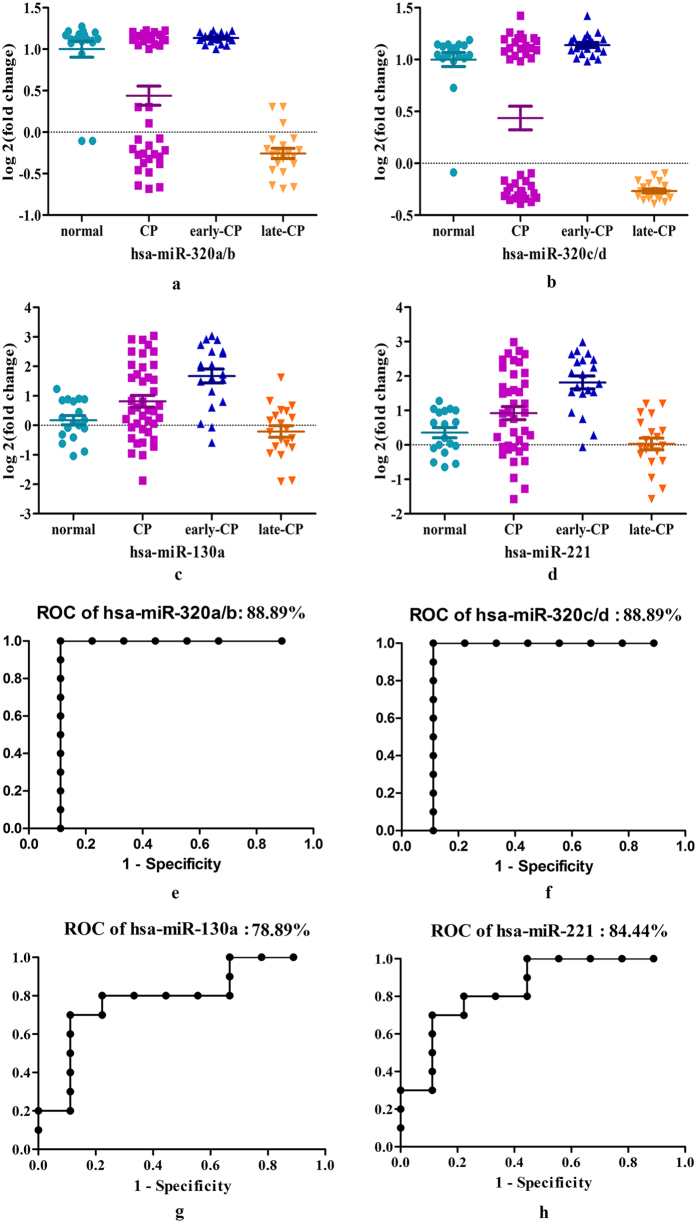
Validation of DEmiRs in a mixed group of patients from clinical practice. Real-time PCR of the selected DEmiRs was performed and statistical significance was assessed with the use of Student’s t-test. (**a**,**b**) Validation of hsa-miR-320a-d by quantitative RT-PCR. The values of each miRNA were normalized; (**c**,**d**) Validation of hsa-miR-130a and hsa-miR-221 by quantitative RT-PCR. The values of each miRNA were normalized; (**e**–**h**) ROC curves of DEmiRs in predicting CP patients (early CP, n = 20; late CP, n = 20; healthy control, n = 18).

Quantitative real-time PCR assays were performed on serum samples from subjects in clinical practice, including early CP patients (n = 20), late CP patients (n = 20) and healthy controls (n = 18). Expression levels of miR-320a/b, miR-320c/d, miR-130a and miR-221were tested. The results showed that as compared to healthy controls, no significant difference was observed in early CP patients on expression levels of miR-320a/b and miR-320c/d, but the late CP patients differentially expressed miR-320a/b and miR-320c/d (Fig. 4a,b). Thus, miR-320a-d might be potential markers for late CP. Compared to healthy controls, the expression levels of miR-130a and miR-221 were upregulated in the early CP patients, however, they were not detected differentially expressed in the late CP patients (Fig. 4c,d).

Moreover, when these 6 miRNAs were respectively applied to predict CP patients in these mixed samples from clinical practice, the results demonstrated that CP patients can be diagnosed effectively (Fig. 4e–h).

### Functional analysis and comparison

In GO Ontology (GO) functional analysis of the DEmiRs, 641 putative targets of DEmiRs were acquired for early CP, which were transformed to 1564 gene IDs successfully and mapped to 6067 GO terms successfully. Finally, 54 significant GO terms were extracted (see Supplementary Table S1 and Supplementary Fig. S1).

In the functional enrichment analysis by multi-GOEAST, there were 4705 putative targets of 141 DEmiRs for late CP, which were mapped to 12250 GO terms successfully. Consequently, 15 shared functional terms were enriched (see Supplementary Table S2) and the DEmiRs in late CP and early CP act on the beta-galactoside alpha-2,6-sialyltransferase activity. The enriched GO terms and their hierarchical relationships in molecular function were displayed with graph (see Supplementary Figs S2 and S3).

Comparing the biological process, 7 common biological processes shared by late CP and early CP were acquired, such as multicellular organismal process, response to stimulus and beta-galactoside alpha-2, 6-sialyltransferase activity etc. However, the miRNAs of early CP mainly regulated the apoptotic process involved in morphogenesis, peptide hormone processing, regulation of programmed cell death etc, while miRNAs of late CP involved in the response to chemical stimulus, pancreatic A cell differentiation and development, immune response and soon.

### Pathway analysis of miRNA targets

There were 28 common pathways regulated by DEmiRs of late CP and early CP (see Supplementary Table S3). In early CP, the DEmiRs were involved in 17 special pathways, while in late CP, the DEmiRs were involved in 12 significant pathways (Tables 1 and 2).

### MiRNA-miRNA correlation network

**Figure 5:**
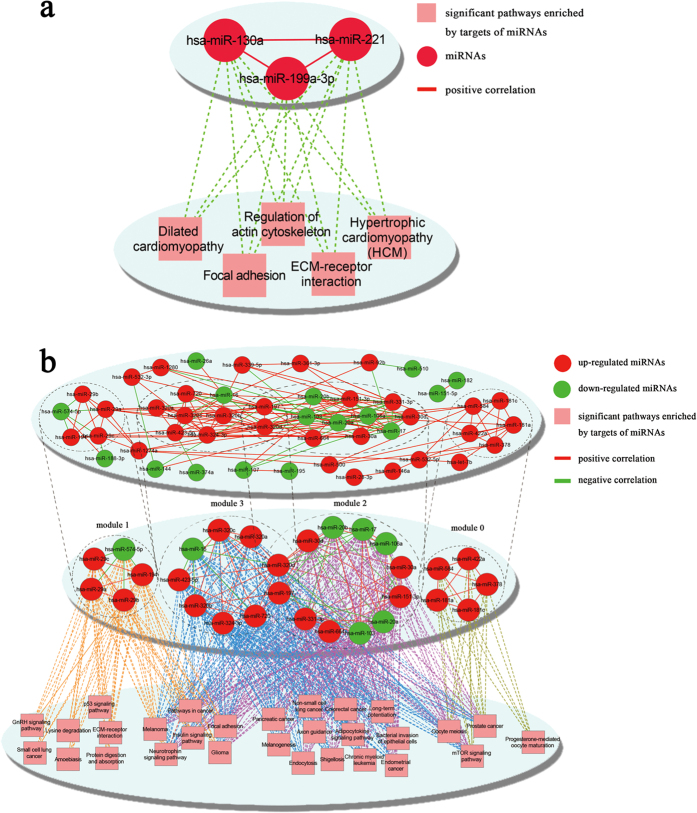
miRNA-miRNA correlation network, functional modules and enriched pathways of early CP and late CP. The miRNA-miRNA correlated network was constructed via the Cytoscape software, where nodes represented miRNA and edges showed their functional synergism. (**a**) Network for early CP. The upper showed miRNA-miRNA correlation network; the lower showed the significant pathways enriched by target genes of miRNAs; (**b**) Network for late CP. The upper displayed miRNA-miRNA correlation network; the middle showed 4 functional modules; the bottom showed significant pathways enriched by target genes of miRNAs in modules.

The correlation analysis and function enrichment obtained 3 correlated miRNA-miRNA pairs for early CP (hsa-miR-199a-3p VS hsa-miR-130a, hsa-miR-221 VS hsa-miR-130a, and hsa-miR-221 VS hsa-miR-199a-3p), all of which were positive correlations with the CE values more than 0.80 (Fig. 5a upper). Hsa-miR-221 and hsa-miR-130a were highly correlated with the highest CE value of 0.90567. MiRNAs in this correlation constructed a functional module and their co-target genes were significantly enriched in the pathways of hypertrophic cardiomyopathy, dilated cardiomyopathy, ECM-receptor interaction, focal adhesion and regulation of actin cytoskeleton (Fig. 5a lower).

For late CP, 160 correlation pairs were obtained, including 45 negative and 115 positive miRNA-miRNA pairs. With the Cytoscape software, the miRNA-miRNA correlation network containing 49 miRNAs and 160 links was constructed (Fig. 5b upper). The topological properties of the miRNA-miRNA correlation network were analyzed (Table 3). Based on the Clique Percolation Method, four highly connected modules were acquired which clustered the DEmiRs in late CP from the miRNA-miRNA correlation network (Table 4, Fig. 5b middle). Pathway enrichment analysis was performed for the co-targeted genes of functional nodules to further understand the miRNA-miRNA correlation network (Fig. 5b bottom).

## Discussion

In the current study, the DEmiRs from the blood of CP patients in GEO database and clinical practice were analyzed. Based on our analysis, the early CP had a distinct miRNA expression pattern that may differentiate it from the healthy and late CP. Moreover, the biomarkers of early CP showed dramatic difference with those of late CP. In fact, much more miRNAs regulating the biological processes were present in the late stage CP patients than the early ones. MiRNA-miRNA correlation network analysis showed that the potential mechanism of the special miRNAs in early CP had its distinguishing features, without overlap with late CP.

CP is characterized by progressive fibrosis and function loss of pancreas^11^. At the late stage of CP, destruction of exocrine and endocrine functions of pancreas would lead to absorptive disorders and diabetes mellitus^12^. The concept of “early CP” was proposed with specific diagnosis criteria, and its clinical value has drawn increasing attention^13^. Early diagnosis and management will improve the effect of treatment and prognosis of patients, but it is a great challenge to distinguish these patients in early stage by routine diagnostic modalities. According to the previous studies about the miRNA in the diagnosis of other pancreatic diseases, we speculated that serum miRNA may be a promising diagnostic marker for early CP.

For the early CP, we identified 4 up-regulated DEmiRs (hsa-miR-199a-3p, hsa-miR-221, hsa-miR-130a and hsa-miR-1471) using agilent miRNA microarrays. Strikingly, miR-130a, miR-199a-3p and miR-221 are closely correlated with each other, functioning as a module to regulate multiple signaling pathways. Previous study showed that up-regulation of miR-199a-3p was associated with liver fibrosis^14^,^15^,^16^,^17^,^18^. A recent serum miRNAs expression profile analysis revealed that hsa-miR-199a-3p was significantly expressed in early pancreatic cancer, suggesting a significant role of miR-199a-3p in the process of origin and development of pancreatic cancer^19^. MiR-130a is demonstrated involved with NF-κB/p65 and PPARγ during liver inflammation^20^, and might also play a key role in chronic periodontitis^21^. These results supported a promising role of miR-130a for the detection of early CP. Regarding to miR-221, recent studies showed that miR-221 was up-regulated during the activation of PSCs^22^, suggesting a role in the pathogenesis of CP. The inflammation-linked miR-221 was found to target Ets-1, which robustly regulated endothelial inflammation, angiogenesis, and vascular remodeling^23^,^24^. In our study, miR-130a and miR-221 were up-regulated in early CP, which may be involved in the pathogenesis of CP.

As for late CP from the GEO database, we found that miR-320a, miR-320b, miR-320c and miR-320d were differentially expressed in late CP. They were highly positively correlated with each other and could predict the late CP patients perfectly. Previous studies have revealed that miR-320 is involved in obesity-related insulin resistance, and inhibition of it improves insulin-PI3K signaling pathways, resulting in increasing insulin sensitivity^25^. A recent study demonstrated that miR-320a was correlated with Ets (v-ets erythroblastosis virus E26 oncogene homolog) family transcription factors^26^,^27^, which control the expression of a variety of pro-fibrotic genes, including the induction of CCN2 by transforming growth factor β^28^. And miR-320b was reported to regulate cell-surface ICAM-1 expression on endothelial cells^29^. Moreover, abnormal expression of miR-320a was found in multiple malignancies including pancreatic cancer^30^,^31^,^32^,^33^ and miR-320c modulated the gemcitabine-resistance of pancreatic cancer cells through SMARCC1^34^. The miR-320s have been shown to be dysregulated in insulin resistance, fibrosis, and pancreatic malignancies, which indicated they played pivotal roles in the development and/or complications of CP. However, further experiment researches are needed to confirm the mechanism of these miRNAs in diverse developmental stages of CP.

To date, a few studies have examined circulating miRNAs in patients with pancreatitis and have sought to identify miRNAs that are predictive for the diagnosis or prognosis of pancreatitis^35^,^36^. However, there is no study about the potential biomarkers for the early diagnosis of CP. In current practice, the diagnosis of early CP is always difficult to be made when the patient had only one onset of acute pancreatitis or abdominal discomfort and it seems the obstacle for investigating serum marker of early CP. In this study, we selected early and late CP patients from our patients cohort prospectively built and all the diagnosis were confirmed according to the follow-up. Herein, we found six noteworthy miRNAs which are markedly increased in the serum of patients. MiR-130a and miR-211 might be promising biomarkers for the detection of early CP, and the miR-320s could predict the late CP. Furthermore, we explored the possibility that biomarker panel for detection of CP though the majority of previous studies only focused on individual miRNAs. In fact, our results showed that single miRNA showed less specificity and sensitivity in predicting early CP, whereas application of both miR-130a and miR-221 was promising for the diagnosis of early CP. Moreover, the biomarker panel of all six miRNAs (miR-130a, miR-221 and miR-320s) may differentiate CP from the health, and detecting the expression profile of miR-320s would benefit the prognosis of CP. In addition, a combination of the miRNA panel based-diagnostic method with approaches such as ultrasound and imaging may significantly improve the diagnostic accuracy. However, these findings need to be validated in larger study populations.

In conclusion, distinct miRNA biomarkers exist in different developmental stages of CP. MiR-130a and miR-221 may play key roles in CP genesis and were potential risk biomarkers in the diagnosis of early CP, while the miR-320a-d could predict the late CP. This panel of 6 serum miRNAs might have the potential to be used clinically for the CP diagnosis, especially the early diagnosis of CP. However, further study is required to verify and ameliorate this diagnosis assay.

## Materials and Methods

### Subjects

The patients with suspected or definite pancreatic disease admitted to the Department of Gastroenterology, Shanghai Changhai Hospital were prospectively collected into electronic database since 2007. CP was diagnosed based on clinical symptoms and imaging morphologic changes^13^ and M-ANNHEIM classification system was adopted to define the clinical stage of CP^37^ (Appendix 1). Stage 1a and 1b (CP patients without pancreatic function insufficiency, with recurrent acute pancreatitis [1a] or chronic abdominal pain [1b]) were defined as early CP and the effectiveness of this definition was proved in previous study with long-term follow-up^38^. Blood samples from 10 patients with early CP and 9 healthy controls with matched ages and sex were collected for miRNA microarrays. Blood samples from another mixed group including early CP (n = 20), late CP (n = 20) patients, and also healthy controls with matched ages and sex (n = 18) were collected for further tests of DEmiRs (see Supplementary Table S4). The study was approved by the Ethics Committee of Shanghai Changhai Hospital and informed consents were obtained from all subjects. All experiments were performed in accordance with the approved guidelines and regulations, including any relevant details.

GSE31568 was the human whole miRNOme project version 1 acquired from GEO (http://www.ncbi.nlm.nih.gov/geo/), which analyzed peripheral blood profiles of controls and patients of 14 different diseases via GPL9040 febit Homo Sapiens miRBase 13.0^39^,^40^. Herein, 38 CP samples (GEO CP) and 38 normal samples from GSE31568 were selected for our study.

### RNA isolation and purification

Peripheral blood (2 ml) was collected in the morning and shifted into EDTA anticoagulant tube quickly blending with pipettes. The plasma was acquired by density-gradient centrifugation and stored at −80 °C. Total RNA was isolated using mirVana™ PARIS™ Kit and stored at −70 °C, and the OD260/OD280 of total RNA were 1.8~2.2, indicating that the RNAs were qualified. The primary endpoints were AUC and expression levels of DEmiRs.

### Expression microarray of CP

We acquired the miRNA expression profile using Agilent miRNA microarrays (G4450AA) carrying 939 individual human miRNA probes. The fluorescence intensity signal values were analyzed with software such as Agilent G4450AA Feature Extraction Software 9.5, Agilent Scan Control Software version A. 7.0 and Agilent Gene Spring software GX9.0 etc.

### DEmiRs

The pre-processing of microarray data was carried out which included the missing value screening, and “limma” package^41^ of R/Bioconductor was to identify the DEmiRs (P < 0.05, |log2 (fold change)| ≥0.8) between CP samples and normal controls. To reduce the false positive results, the Benjamini Hochberg (BH) method was used to adjust the p-values into false discovery rate (FDR) and the FDR < 0.05 was selected as cut-off criterion to identify DEmiRs. To measure the diagnostic value of these DEmiRs, we designed a radial based function (RBF) kernel-based SVM and then utilized 5-fold cross-validation strategy to evaluate the performance of SVM classifier. The outcome of prediction was then assessed by receiver operating characteristic (ROC) curve analysis.

### Quantitative real-time PCR for miRNA

Real-time PCR of the selected DEmiRs was performed using LightCycler^®^ 480 II Real-time PCR Instrument (Roche, Swiss). The miRNA-specific primer sequences were designed by the manufacturer (Life Technologies) based on the miRNA sequences obtained from the miRBase database, and the miRNA-specific primer sequences were listed in Supplementary Table S5.

### Functional enrichment analysis of DEmiR target genes

By integrating miRanda^42^, PicTar^43^,^44^, TargetScan^45^, DIANA-microT^46^, miRecord^47^, miRbase^48^ and targetMiner^49^ databases, the targets that existed in more than 3 databases were selected as the target genes of DEmiRs in early and late CP, respectively. For the target genes of DEmiRs, a GO enrichment analysis was performed to identify GO terms with higher confidence with GOEAST^50^. By default, GOEAST uses hypergeometric test to assess the over-represented for GO term in biological process, cellular components and molecular function categories with p-value < 0.05. The risk pathways were the ones where most of the genes were likely differentially expressed and were identified using the Database for Annotation, Visualization and Integrated Discovery (DAVID) tool^51^. Furthermore, the gene distribution from the unigene database of NCBI (http://www.ncbi.nlm.nih.gov/unigene) was downloaded and then analyzed for early and late CP.

### Pathway enrichment analysis

All pathways from Kyoto Encyclopedia of Genes and Genomes (KEGG) database are considered for analysis, which were organized in hierarchical structure. In our study, based on closeness of genes in pathways through a given distance parameter, the iSubpathwayMiner R package was applied to identify the significantly miRNA regulated subpathways.

### Construction of miRNA-miRNA correlated network

To study the synergism of miRNAs for further exploration of miRNA functions in different stages of CP, the miRNA-miRNA pairs for the DEmiRs were acquired randomly, and their co-target genes were identified. Then, the enrichment analysis of pathways by hypergeometric distribution was performed. For a given miRNA-miRNA pair, the probability p-value in pathway term *i* was calculated according to the following equation:


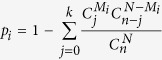


where *N* is the number of all targets, *M*_*i*_ is the total number of genes in pathway term *i, n* is the size of all co-targets, and *k* is the number of co-targets that are also annotated to pathway term *i*. The p-value was further adjusted to FDR by BH method and the pathway terms with p-value < 0.05 are significant. After performing the function enrichment, the miRNA functional synergistic pairs was constructed if the miRNA-miRNA pair co-targets at least one pathway terms. Moreover, based on the miRNA expression profile, the correlated miRNA pairs were selected by Pearson’s correlation coefficient where the pairs with CE > 0 were positive correlation and CE < 0 were the negative correlation. The miRNA-miRNA correlated network was constructed via the Cytoscape software, where nodes represented miRNA and edges showed their functional synergism.

## Additional Information

**How to cite this article**: Xin, L. *et al*. Novel blood-based microRNA biomarker panel for early diagnosis of chronic pancreatitis. *Sci. Rep.* **7**, 40019; doi: 10.1038/srep40019 (2017).

**Publisher's note:** Springer Nature remains neutral with regard to jurisdictional claims in published maps and institutional affiliations.

## Supplementary Material

Supplementary Files

## Figures and Tables

**Table 1:** Special pathways regulated by DEmiRs of early CP.

pathway ID	pathway Name	annMoleculeRatio	pvalue	fdr
path:00310	Lysine degradation	26/7523	0.0007324	0.0053467
path:00640	Propanoate metabolism	19/7523	0.0034723	0.0190111
path:04115	p53 signaling pathway	37/7523	0.0004022	0.0033879
path:04270	Vascular smooth muscle contraction	52/7523	0.0090753	0.0441665
path:04350	TGF-beta signaling pathway	46/7523	4.99E-05	0.0008017
path:04520	Adherens junction	43/7523	3.80E-06	0.0001386
path:04530	Tight junction	68/7523	3.09E-05	0.0007518
path:04670	Leukocyte transendothelial migration	61/7523	4.70E-05	0.0008017
path:04710	Circadian rhythm - mammal	17/7523	5.13E-05	0.0008017
path:04720	Long-term potentiation	39/7523	0.0002213	0.0024232
path:04920	Adipocytokine signaling pathway	37/7523	0.0001765	0.002148
path:04971	Gastric acid secretion	37/7523	0.0023473	0.0138932
path:05110	Vibrio cholerae infection	30/7523	0.0012145	0.0078229
path:05210	Colorectal cancer	34/7523	0.0011758	0.0078229
path:05211	Renal cell carcinoma	43/7523	1.24E-06	6.80E-05
path:05216	Thyroid cancer	19/7523	0.0006425	0.0048524
path:05414	Dilated cardiomyopathy	42/7523	0.0032348	0.0181644

**Table 2:** Special pathways regulated by DEmiRs of late CP.

pathway ID	pathway Name	annMoleculeRatio	pvalue	fdr
path:04660	T cell receptor signaling pathway	103/19312	0.0003111	0.0058564
path:05142	Chagas disease	101/19312	0.0003828	0.005988
path:00240	Pyrimidine metabolism	97/19312	0.0005782	0.0079147
path:04062	Chemokine signaling pathway	178/19312	0.0013869	0.0175316
path:04080	Neuroactive ligand-receptor interaction	255/19312	0.0028213	0.0236738
path:04640	Hematopoietic cell lineage	79/19312	0.0035589	0.026889
path:00230	Purine metabolism	153/19312	0.0038001	0.0277408
path:05410	Hypertrophic cardiomyopathy	76/19312	0.0047828	0.0337882
path:04960	Aldosterone-regulated sodium reabsorption	42/19312	0.006176	0.0409864
path:04664	Fc epsilon RI signaling pathway	73/19312	0.0064117	0.0412991
path:05219	Bladder cancer	40/19312	0.0078708	0.0478804
path:05412	Arrhythmogenic right ventricular cardiomyopathy (ARVC)	69/19312	0.0094385	0.0516759

**Table 3:** Global topological properties of the miRNA-miRNA correlation network for late CP.

Average Shortest Path Length	Betweenness Centrality	Closeness Centrality	Clustering Coefficient	Degree
2.673	0.037	0.403	0.475	6.531

**Table 4:** Functional modules in miRNA-miRNA correlation network of late CP.

Module	miRNAs
0	hsa-miR-181a hsa-miR-181c hsa-miR-378 hsa-miR-422a hsa-miR-584
1	hsa-miR-29a hsa-miR-194 hsa-miR-29b hsa-miR-29c hsa-miR-574-5p
2	hsa-miR-103 hsa-miR-151-3p hsa-miR-106a hsa-miR-17 hsa-miR-197 hsa-miR-20a hsa-miR-20b hsa-miR-30a hsa-miR-30d hsa-miR-320d hsa-miR-331-3p hsa-miR-664
3	hsa-miR-197 hsa-miR-16 hsa-miR-320a hsa-miR-320b hsa-miR-320c hsa-miR-320d hsa-miR-324-3p hsa-miR-423-5p hsa-miR-720
