# Supplementary material for: Novel blood-based microRNA biomarker panel for early diagnosis of chronic pancreatitis
Source: Sci Rep. 2017 Jan 11;7:40019. doi: 10.1038/srep40019 (PMC5225423; doi:10.1038/srep40019)
Supplement: Supplementary Files [file srep40019-s1.pdf]

Supplementary Files

Novel blood-based microRNA biomarker panel for early diagnosis of chronic pancreatitis

Lei Xin[1]*, M.D., Jun Gao[1]*, Ph.D., Dan Wang[1]*, M.D., Jin-Huan Lin[1], M.D., Zhuan Liao[1], M.D., Jun-Tao Ji[1], M.D., Ting-Ting Du[1], M.D., Fei Jiang[1], M.D., Liang-Hao Hu[1], M.D., Zhao-Shen Li[1], M.D.

[1]Department of Gastroenterology, Changhai Hospital, the Second Military Medical University, Shanghai, China

* Dr. Xin, Dr. Gao and Dr. Wang contributed equally to this work.

Supplementary Tables

GOID	Ontology	Term	P-value
GO:0003835	MF	beta-galactoside alpha-2,6-sialyltransferase activity	2.69E-15
GO:0019778	MF	APG12 activating enzyme activity	1.64E-06
GO:0008234	MF	cysteine-type peptidase activity	3.17E-06
GO:0004839	MF	ubiquitin activating enzyme activity	3.18E-06
GO:0034596	MF	phosphatidylinositol phosphate 4-phosphatase activity	4.94E-06
GO:0003906	MF	DNA-(apurinic or apyrimidinic site) lyase activity	1.12E-05
GO:0008373	MF	sialyltransferase activity	2.94E-05
GO:0044020	MF	histone methyltransferase activity (H4-R3 specific)	8.37E-05
GO:0016765	MF	transferase activity, transferring alkyl or aryl (other than methyl) groups	0.000152
GO:0016316	MF	phosphatidylinositol-3,4-bisphosphate 4-phosphatase activity	0.000358
GO:0034593	MF	phosphatidylinositol bisphosphate phosphatase activity	0.0007102
GO:0019104	MF	DNA N-glycosylase activity	0.0009819
GO:0008281	MF	sulfonylurea receptor activity	0.0009956
GO:0042611	CC	MHC protein complex	3.17E-06
GO:0030870	CC	Mre11 complex	4.94E-06
GO:0030427	CC	site of polarized growth	5.90E-06
GO:0044421	CC	extracellular region part	9.66E-06
GO:0005576	CC	extracellular region	1.12E-05
GO:0030426	CC	growth cone	1.15E-05
GO:0044459	CC	plasma membrane part	8.11E-05
GO:0044295	CC	axonal growth cone	9.83E-05
GO:0030016	CC	myofibril	0.000288
GO:0043292	CC	contractile fiber	0.0002918
GO:0005615	CC	extracellular space	0.000358
GO:0005744	CC	mitochondrial inner membrane presequence translocase complex	0.000358
GO:0032501	BP	multicellular organismal process	1.97E-06
GO:0006305	BP	DNA alkylation	3.17E-06
GO:0006306	BP	DNA methylation	3.17E-06

GO:0060561	BP	apoptotic process involved in morphogenesis	9.66E-06
GO:0016486	BP	peptide hormone processing	1.15E-05
GO:0043067	BP	regulation of programmed cell death	1.47E-05
GO:0042981	BP	regulation of apoptotic process	1.56E-05
GO:0072023	BP	thick ascending limb development	2.94E-05
GO:0072233	BP	metanephric thick ascending limb development	2.94E-05
GO:0050896	BP	response to stimulus	2.99E-05
GO:0010623	BP	developmental programmed cell death	3.27E-05
GO:0044728	BP	DNA methylation or demethylation	3.51E-05
GO:0007588	BP	excretion	5.45E-05
GO:0044707	BP	single-multicellular organism process	6.82E-05
GO:0010941	BP	regulation of cell death	7.68E-05
GO:0006914	BP	autophagy	0.0001079
GO:0021859	BP	pyramidal neuron differentiation	0.0001379
GO:0021860	BP	pyramidal neuron development	0.0001379
GO:0072021	BP	ascending thin limb development	0.0001977
GO:0072218	BP	metanephric ascending thin limb development	0.0001977
GO:0003407	BP	neural retina development	0.0002895
GO:0006304	BP	DNA modification	0.0003002
GO:0003285	BP	septum secundum development	0.000358
GO:0050808	BP	synapse organization	0.000425
GO:0051491	BP	positive regulation of filopodium assembly	0.000425
GO:0051023	BP	regulation of immunoglobulin secretion	0.0005942
GO:0000244	BP	assembly of spliceosomal tri-snRNP	0.000743
GO:0072236	BP	metanephric loop of Henle development	0.000743
GO:0051489	BP	regulation of filopodium assembly	0.0007666

*BP: biological_process; CC; CC: cellular_component; MF: molecular_function

Supplementary Table S1. GO functional enrichment for the targets of DEmiRs in early CP

GOID	Ontology	Term	Level	MIN-p-value	MAX-p-value	AVG-p-value
GO:0042611	CC	MHC protein complex	6	1.6066E-33	3.167E-06	1.583E-06
GO:0044421	CC	extracellular region part	2	5.22874E-85	9.662E-06	4.831E-06
GO:0005576	CC	extracellular region	1	1.59705E-94	1.121E-05	5.604E-06
GO:0044459	CC	plasma membrane part	5	8.06328E-69	8.113E-05	4.056E-05
GO:0030016	CC	myofibril	8	1.85948E-31	0.000288	0.000144
GO:0043292	CC	contractile fiber	7	1.24842E-28	0.0002918	0.0001459
GO:0005615	CC	extracellular space	3	7.89242E-53	0.000358	0.000179
GO:0032501	BP	multicellular organismal process	1	3.81019E-74	1.967E-06	9.834E-07
GO:0050896	BP	response to stimulus	1	7.80417E-63	2.99E-05	1.495E-05
GO:0044707	BP	single-multicellular organism process	2	4.25127E-69	6.816E-05	3.408E-05
GO:0007588	BP	excretion	4	2.14248E-05	5.45E-05	3.796E-05
GO:0003407	BP	neural retina development	10	2.32321E-05	0.0002895	0.0001563
GO:0006305	BP	DNA alkylation	8	3.16666E-06	0.0003543	0.0001787
GO:0006306	BP	DNA methylation	9	3.16666E-06	0.0003543	0.0001787
GO:0003835	MF	beta-galactoside alpha-2,6-sialyltransferase activity	5	2.6925E-15	2.002E-07	1.001E-07

*BP: biological_process; CC; CC: cellular_component; MF: molecular_function

Supplementary Table S2. Common GO terms regulated by DEmiRs in late CP and early CP

pathwayId	pathwayName
path:00071	Fatty acid metabolism
path:04010	MAPK signaling pathway
path:04012	ErbB signaling pathway
path:04020	Calcium signaling pathway
path:04120	Ubiquitin mediated proteolysis
path:04141	Protein processing in endoplasmic reticulum
path:04142	Lysosome
path:04144	Endocytosis
path:04150	mTOR signaling pathway
path:04210	Apoptosis
path:04310	Wnt signaling pathway
path:04360	Axon guidance
path:04510	Focal adhesion
path:04666	Fc gamma R-mediated phagocytosis
path:04722	Neurotrophin signaling pathway
path:04810	Regulation of actin cytoskeleton
path:04910	Insulin signaling pathway
path:04916	Melanogenesis
path:05100	Bacterial invasion of epithelial cells
path:05120	Epithelial cell signaling in Helicobacter pylori infection
path:05200	Pathways in cancer
path:05212	Pancreatic cancer
path:05214	Glioma
path:05215	Prostate cancer
path:05218	Melanoma
path:05220	Chronic myeloid leukemia
path:05222	Small cell lung cancer
path:05223	Non-small cell lung cancer

Supplementary Table S3. Common pathways regulated by DEmiRs of late CP and early CP.

miRNAs	Primers	Sequences
**Control**	P1-F	TGAGAAGTCCGTCACACACCA
	P1-R	TAAGTCGTCTGTCAGCAGCAC
**hsa-miR-130a**	h-P130a-F	TGAGAAGTCCGTCACACACCA
	h-P130a-R	GGCGACAGTGCAATGTTAA
**hsa-miR-221**	h-P221-3p-F	AGTCACATCTACATTGTCTGC
	h-P221-3p-R	TGAGAAGTCCGTCACACACCA
**hsa-miR-320a/b**	h-P320a/b-F	CACAAAAGCTGGGTTGAGAGG
	h-P320-R	TGAGAAGTCCGTCACACACCA
**hsa-miR-320c/d**	h-P320c/d-F	CAGTAGGAAAAGCTGGGTTGAG
	h-P320-R	TGAGAAGTCCGTCACACACCA

Supplementary Table S4. List of miRNA-specific primer sequences

Appendix 1. M-ANNHEIM clinical staging of chronic pancreatitis.

Asymptomatic chronic pancreatitis

Stage 0 of subclinical chronic pancreatitis

a. Period without symptoms (determination by chance, eg, autopsy)

b. Acute pancreatitisVsingle episode (possible onset of chronic pancreatitis)

c. Acute pancreatitis with severe complications

Symptomatic chronic pancreatitis

Stage 1 without pancreatic insufficiency

a. (Recurrent) acute pancreatitis (no pain between episodes of acute pancreatitis)

b. Recurrent or chronic abdominal pain (including pain between episodes of acute pancreatitis)

c. 1a/b with severe complications

Stage 2 of partial pancreatic insufficiency

a. Isolated exocrine (or endocrine) pancreatic insufficiency (without pain)

b. Isolated exocrine (or endocrine) pancreatic insufficiency (with pain)

c. 2a/b with severe complications

Stage 3 of painful complete pancreatic insufficiency

a. Exocrine and endocrine insufficiency (with pain, e.g. requiring pain medication)

b. 3a with severe complications

Stage 4 of secondary painless disease (burnout)

a. Exocrine and endocrine insufficiency without pain and without severe complications

b. Exocrine and endocrine insufficiency without pain and with severe complications

Supplementary Figures Legends

Supplementary Figure S1. GO functional enrichment for the targets of DEmiRs in early CP

Supplementary Figure S2. The enriched GO terms of DEmiRs in early CP and late CP and their hierarchical relationships in molecular function

Supplementary Figure S3. The enriched GO terms of DEmiRs in early CP and late CP and their hierarchical relationships in molecular function

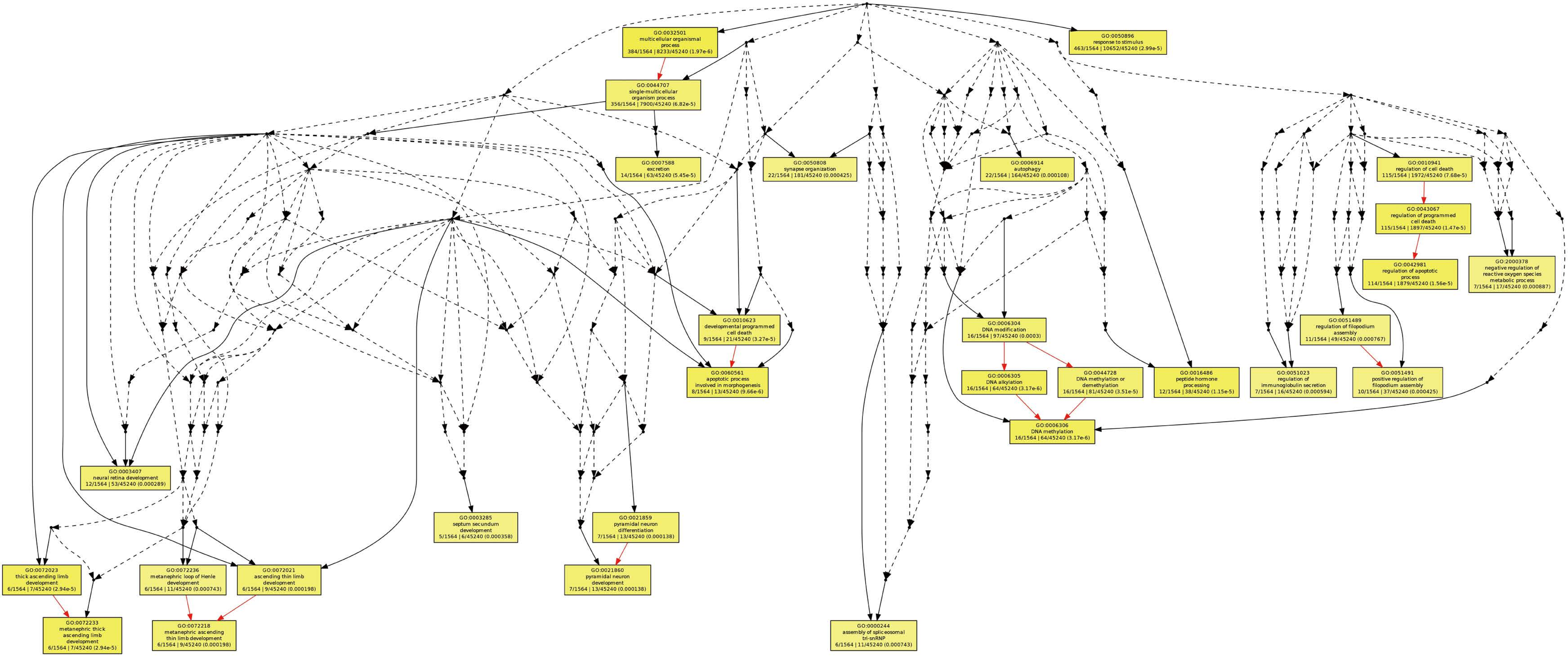
GO:0032501
multicellular organismal
process
384/1564 | 8233/45240 (1.97e-6)
GO:0050896
response to stimulus
463/1564 | 10652/45240 (2.99e-5)
GO:0044707
single-multicellular
organism process
356/1564 | 7900/45240 (6.82e-5)
GO:0007588
excretion
14/1564 | 63/45240 (5.45e-5)
GO:0050808
synapse organization
22/1564 | 181/45240 (0.000425)
GO:0006914
autophagy
22/1564 | 164/45240 (0.000108)
GO:0010941
regulation of cell death
115/1564 | 1972/45240 (7.68e-5)
GO:0043067
regulation of programmed
cell death
115/1564 | 1897/45240 (1.47e-5)
GO:0042981
regulation of apoptotic
process
114/1564 | 1879/45240 (1.56e-5)
GO:2000378
negative regulation of
reactive oxygen species
metabolic process
7/1564 | 17/45240 (0.000887)
GO:0010623
developmental programmed
cell death
9/1564 | 21/45240 (3.27e-5)
GO:0060561
apoptotic process
involved in morphogenesis
8/1564 | 13/45240 (9.66e-6)
GO:0006304
DNA modification
16/1564 | 97/45240 (0.0003)
GO:0006305
DNA alkylation
16/1564 | 64/45240 (3.17e-6)
GO:0044728
DNA methylation or
demethylation
16/1564 | 81/45240 (3.51e-5)
GO:0016486
peptide hormone
processing
12/1564 | 38/45240 (1.15e-5)
GO:0051489
regulation of filopodium
assembly
11/1564 | 49/45240 (0.000767)
GO:0051023
regulation of
immunoglobulin secretion
7/1564 | 16/45240 (0.000594)
GO:0051491
positive regulation of
filopodium assembly
10/1564 | 37/45240 (0.000425)
GO:0006306
DNA methylation
16/1564 | 64/45240 (3.17e-6)
GO:0003407
neural retina development
12/1564 | 53/45240 (0.000289)
GO:0003285
septum secundum
development
5/1564 | 6/45240 (0.000358)
GO:0021859
pyramidal neuron
differentiation
7/1564 | 13/45240 (0.000138)
GO:0021860
pyramidal neuron
development
7/1564 | 13/45240 (0.000138)
GO:0072023
thick ascending limb
development
6/1564 | 7/45240 (2.94e-5)
GO:0072236
metanephric loop of Henle
development
6/1564 | 11/45240 (0.000743)
GO:0072021
ascending thin limb
development
6/1564 | 9/45240 (0.000198)
GO:0072233
metanephric thick
ascending limb
development
6/1564 | 7/45240 (2.94e-5)
GO:0072218
metanephric ascending
thin limb development
6/1564 | 9/45240 (0.000198)
GO:0000244
assembly of spliceosomal
tri-snRNP
6/1564 | 11/45240 (0.000743)

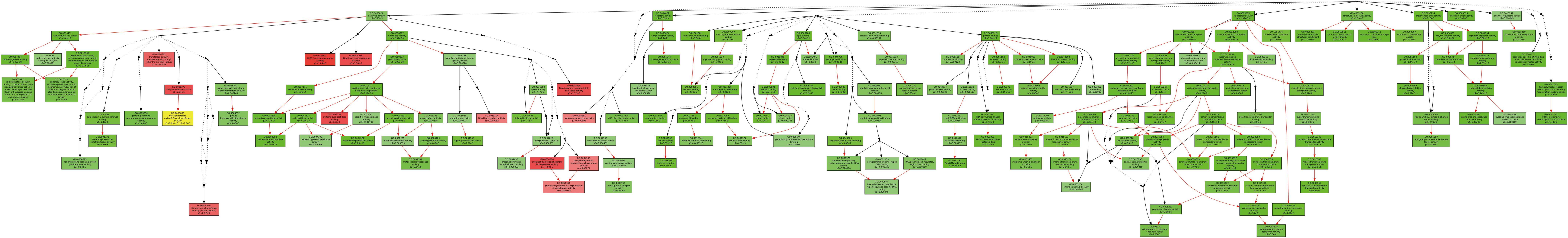

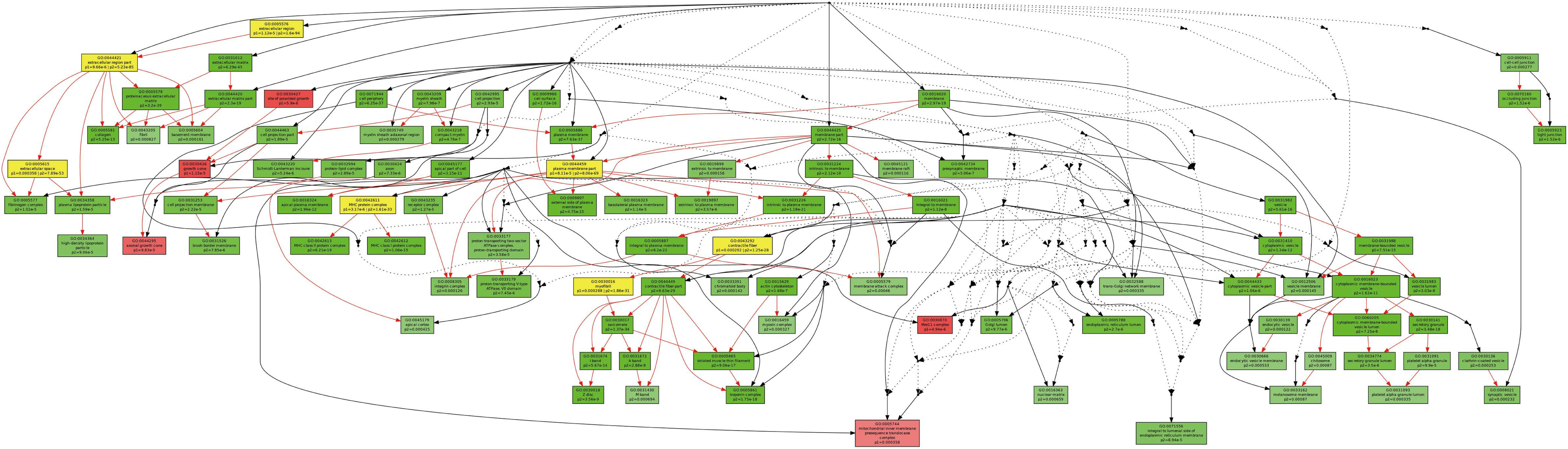

GO:0005576 extracellular region p1=1.12e-5 | p2=1.6e-94
GO:0044421 extracellular region part p1=9.66e-6 | p2=5.23e-85
GO:0031012 extracellular matrix p2=6.29e-45
GO:0005911 cell-cell junction p2=0.000277
GO:0044420 extracellular matrix part p2=2.3e-19
GO:0030427 site of polarized growth p1=5.9e-6
GO:0071944 cell periphery p2=6.25e-37
GO:0043209 myelin sheath p2=7.98e-7
GO:0042995 cell projection p2=2.93e-5
GO:0009986 cell surface p2=1.72e-16
GO:0016020 membrane p2=2.97e-19
GO:0070160 occluding junction p2=1.52e-6
GO:0005578 proteinaceous extracellular matrix p2=3.2e-39
GO:0005581 collagen p2=5.25e-13
GO:0043205 fibril p2=0.000827
GO:0005604 basement membrane p2=0.000161
GO:0035749 myelin sheath adaxonal region p2=0.000279
GO:0043218 compact myelin p2=4.76e-7
GO:0005886 plasma membrane p2=7.63e-37
GO:0044425 membrane part p2=2.72e-16
GO:0005923 tight junction p2=1.52e-6
GO:0005615 extracellular space p1=0.000358 | p2=7.89e-53
GO:0044463 cell projection part p2=1.89e-5
GO:0030426 growth cone p1=1.15e-5
GO:0043220 Schmidt-Lanterman incisure p2=5.24e-6
GO:0032994 protein-lipid complex p2=2.89e-5
GO:0030424 axon p2=7.33e-6
GO:0045177 apical part of cell p2=3.15e-11
GO:0044459 plasma membrane part p1=8.11e-5 | p2=8.06e-69
GO:0019898 extrinsic to membrane p2=0.000158
GO:0031224 intrinsic to membrane p2=2.12e-10
GO:0045121 membrane raft p2=0.000116
GO:0042734 presynaptic membrane p2=5.06e-7
GO:0005577 fibrinogen complex p2=1.02e-5
GO:0034358 plasma lipoprotein particle p2=1.59e-5
GO:0031253 cell projection membrane p2=2.22e-5
GO:0016324 apical plasma membrane p2=1.96e-12
GO:0042611 MHC protein complex p1=3.17e-6 | p2=1.61e-33
GO:0043235 receptor complex p2=1.27e-5
GO:0009897 external side of plasma membrane p2=4.75e-15
GO:0016323 basolateral plasma membrane p2=1.14e-5
GO:0019897 extrinsic to plasma membrane p2=3.57e-6
GO:0031226 intrinsic to plasma membrane p2=1.18e-21
GO:0016021 integral to membrane p2=1.12e-8
GO:0031982 vesicle p2=5.61e-16
GO:0034364 high-density lipoprotein particle p2=9.06e-5
GO:0044295 axonal growth cone p1=9.83e-5
GO:0031526 brush border membrane p2=7.85e-6
GO:0042613 MHC class II protein complex p2=6.21e-19
GO:0042612 MHC class I protein complex p2=1.06e-15
GO:0033177 proton-transporting two-sector ATPase complex, proton-transporting domain p2=3.58e-5
GO:0005887 integral to plasma membrane p2=8.2e-22
GO:0043292 contractile fiber p1=0.000292 | p2=1.25e-28
GO:0031410 cytoplasmic vesicle p2=1.24e-12
GO:0031988 membrane-bounded vesicle p2=7.51e-15
GO:0008305 integrin complex p2=0.000126
GO:0033179 proton-transporting V-type ATPase, V0 domain p2=7.45e-6
GO:0030016 myofibril p1=0.000288 | p2=1.86e-31
GO:0044449 contractile fiber part p2=6.63e-29
GO:0033391 chromatoid body p2=0.000142
GO:0015629 actin cytoskeleton p2=1.68e-7
GO:0005579 membrane attack complex p2=0.00046
GO:0032588 trans-Golgi network membrane p2=0.000335
GO:0044433 cytoplasmic vesicle part p2=1.06e-6
GO:0012506 vesicle membrane p2=0.000145
GO:0016023 cytoplasmic membrane-bounded vesicle p2=1.62e-11
GO:0031983 vesicle lumen p2=3.03e-8
GO:0045179 apical cortex p2=0.000425
GO:0030017 sarcomere p2=1.37e-34
GO:0016459 myosin complex p2=0.000327
GO:0030870 Mre11 complex p1=4.94e-6
GO:0005796 Golgi lumen p2=9.77e-6
GO:0005788 endoplasmic reticulum lumen p2=2.7e-6
GO:0030139 endocytic vesicle p2=0.000122
GO:0060205 cytoplasmic membrane-bounded vesicle lumen p2=7.25e-8
GO:0030141 secretory granule p2=3.48e-18
GO:0031674 I band p2=5.67e-14
GO:0031672 A band p2=2.88e-9
GO:0005865 striated muscle thin filament p2=9.06e-17
GO:0030666 endocytic vesicle membrane p2=0.000533
GO:0045009 chitosome p2=0.00087
GO:0034774 secretory granule lumen p2=3.5e-6
GO:0031091 platelet alpha granule p2=9.9e-5
GO:0030136 clathrin-coated vesicle p2=0.000253
GO:0030018 Z disc p2=3.56e-9
GO:0031430 M band p2=0.000694
GO:0005861 troponin complex p2=1.75e-18
GO:0016363 nuclear matrix p2=0.000659
GO:0033162 melanosome membrane p2=0.00087
GO:0031093 platelet alpha granule lumen p2=0.000335
GO:0008021 synaptic vesicle p2=0.000232
GO:0005744 mitochondrial inner membrane presequence translocase complex p1=0.000358
GO:0071556 integral to lumenal side of endoplasmic reticulum membrane p2=8.94e-5
